# *AfLFY*, a *LEAFY* homolog in *Argyranthemum frutescens*, controls flowering time and leaf development

**DOI:** 10.1038/s41598-020-58570-x

**Published:** 2020-01-31

**Authors:** Jing Hu, Qi Jin, Yueping Ma

**Affiliations:** College of Life and Health Sciences, Northeastern University, Shenyang, 110004 China

**Keywords:** Plant breeding, Plant development

## Abstract

Flowering is important for plant propagation and survival, and it is also closely related to human life. Identifying the molecular mechanisms underlying flower development is essential for plant improvement and breeding. Flower development is a complex physiological process that is regulated by multiple genes. *LFY* genes play important roles in the floral meristem transition and act as crucial integrators in regulating the floral gene network. *Argyranthemum frutescens* is an ornamental species cultivated for floral displays, yet little is known about molecular mechanisms driving its flower development. In this study, the *LEAFY* gene homologue, *AfLFY*, was identified and cloned from *A*. *frutescens*, and its role and expression patterns were characterized. Two distinct copies of *AfLFY* were found in the *A*. *frutescens* genome and both sequences contained a 1248 bp open reading frame that encoded 415 amino acids. The putative protein sequences have a typical LFY family domain. In addition, *AfLFY* was expressed at the highest levels in young leaves of the vegetative stage and in the shoot apical bud meristem of the reproductive stage. Phylogenetic analysis showed that *AfLFY* was most closely related to DFL from *Chrysanthemum lavandulifolium*. Subcellular localization studies revealed that AfLFY localized to the nucleus. Heterologous expression of *AfLFY* in transgenic tobacco plants shortened its period of vegetative growth, converted the lateral meristems into terminal flowers and promoted precocious flowering. In addition, transgenic plants exhibited obvious morphological changes in leaf shape. qRT-PCR analysis indicated that the expression levels genes related to flowering, *FT*, *SOC1*, and *AP1* were significantly upregulated in *AfLFY* transgenic plants. Our findings suggested that the *AfLFY* gene plays a vital role in promoting flowering and leaf development in *A*. *frutescens*. These results laid a foundation for us to understand the mechanism of *AfLFY* in regulation flowering, and the results will be helpful in improving *A*. *frutescens* through molecular breeding.

## Introduction

Flowering is a vital component of the plant life cycle that influences the success of plant reproduction. The transition from vegetative to reproductive development occurs in response to various endogenous and exogenous cues, and these cues are integrated by a complex molecular network^1–3^. Many floral integrator genes are involved in this molecular network, such as *FLOWERING LOCUST* (*FT*) and *SUPPRESSOR OF OVEREXPRESSION OF CONSTANS 1* (*SOC1*), which work with the floral meristem identity gene *LEAFY* (*LFY*), to initiate the growth of floral meristems^4–7^. *CONSTANS* (*CO*) mediates the floral process in the photoperiod pathway and activates *LFY* expression directly or indirectly through other floral integrators^8–10^.

*LFY* genes were initially described as floral meristem identity genes in Antirrhinum and Arabidopsis^11,12^, and were later shown to act as genetic switches directing the transition from inflorescence meristems to flower meristems^13^. Overexpression of *LFY* homologs stimulated flower initiation in both dicotyledonous and monocotyledonous species^14–17^. *LFY* is thought to act as a signaling gateway, integrating signals from global floral pathway processes and activating downstream *ABC* genes that specify unique floral meristem and organ identities^18–23^. For example, *APETALA1* (*AP1*), which determines floral meristem and organ identities in *Arabidopsis*, is directly activated by *LFY*^24–27^.

*LFY* homologs have been identified among distantly related species^28–35^. LFY proteins from most species share conserved regions, such as a proline-rich region, a leucine zipper, an acidic region, and a basic region formed by an arginine core and lysine residues; however, in some cases the protein structures and gene expression patterns differ among species. For example, the proline-rich region is absent in gymnosperms, eucalyptus, cotton, and papaya, among others^36–40^. *FLO* in *Antirrhinum majus* was only found to be expressed in the reproductive phase. Expression of *GhLFY* in *Gerbera hybrida* is restricted to the reproductive transformation phase and to early flower development^41^. Although associated with flowering, low levels of *LFY* transcripts were also detected in vegetative tissues during the vegetative growth in some plant species^33–35,38,41–45^. *LFY* expression patterns in rice and wheat differed from those of other *LFY* homologs. In rice, *RFL* transcripts were detected in the initiation of the floral meristem much later than in developing branches and young panicle roots, and no transcripts were found in mature leaves^46^. In wheat, *WFL* transcripts were observed in all layers of the young spike except in the spikelet initiation sites, axillary meristem, and developing palea^47^. Identification and characterization of additional *LFY* homologs is needed to understand the evolution and functions of *LFY* and its specific motifs in flowering regulation.

*LFY*-like genes from *Chrysanthemum* are highly expressed in the flower bud^33,34^, which suggests that *Chrysanthemum LFY* genes may play important roles in the transition from vegetative to reproductive meristems; However, their functions remain unclear due to a lack of functional investigation. *Argyranthemum frutescens* (Asteraceae) is a popular cultivated species that is used globally as a potted plant, ground cover, or a garden ornamental for its foliage, flower color^48,49^ and long flowering period. However, the current research on *A*. *frutescens* has mostly focused on medicinal purposes, pathogen invasion and plant diseases^50–55^, and the genetic mechanisms underlying floral development in *A*. *frutescens* are not well known. Here, the *LFY* homolog *AfLFY* was identified and characterized in *A*. *frutescens*. The expression patterns of *AfLFY* in different tissues and organs were examined by quantitative real-time PCR (qRT-PCR). The protein subcellular localization was investigated by transient expression in onion epidermal cells. The function of *AfLFY* was explored by studying heterologous expression in *Nicotiana tabacum L* (tobacco). These studies contribute to understanding the molecular mechanisms of *AfLFY* in regulating floral development and will be helpful for molecular breeding of *A*. *frutescens*.

## Materials and Methods

### Plant material

*A*. *frutescens* plants used in this study were collected from Shennongjia in Hubei Province, China, and were planted in the nursery garden of Northeastern University, China. Plant tissues for RNA extraction were collected at the relevant developmental stages and were immediately frozen in liquid nitrogen before storing at −80 °C. Total RNA was extracted using a Plant RNA kit (Omega, USA) and then was treated with DNase I (Omega, USA) to remove genomic DNA. Genomic DNA was isolated from fresh leaves using the CTAB method described by Couch and Fritz with minor modifications^56^.

### Cloning *AfLFY*

First-strand cDNA was synthesized from a library of inflorescence shoot apices using a Revert Aid First Strand cDNA Synthesis kit (Thermo, USA). Full-length *AfLFY* cDNA was obtained by RT-PCR with primers AfLFY–F and AfLFY–R, which were designed against *LFY* homolog from chrysanthemum (Table 1). PCR amplifications was performed in 25 µL reaction volumes containing 1.5 μL of cDNA, 0.3 μL of LA Taq polymerase (TaKaRa, Japan), 2.5 μL of 10 × LA Buffer, 2.0 μL of dNTPs, 1.0 µL of each primer, and 16.7 μL of ddH_2_O. Thermocycling conditions were as follows: denaturation for 5 min at 95 °C; 35 cycles of 95 °C for 50 s, annealing at 55 °C for 30 s, and extension at 72 °C for 90 s; and final extension at 72 °C for 10 min. PCR products were assessed using 1% agarose gel electrophoresis and then were purified using a Trace agarose gel DNA recovery kit (Zhongmeitaihe, Beijing China). Purified PCR products were cloned and transformed into *E*. *coli* using a pCloneEZ-TA-Amp/HC Cloning kit (Thermo, USA). Transformed colonies were identified by PCR with gene-specific primers and restriction digestion, and six positive clones were confirmed by sequencing (Zhongmeitaihe Gene Company, Beijing, China).

**Table 1:** Primers used in this study.

Primer name	Primer sequence (5′ → 3′)	Application
AfLFY –F	GTGGATCCATGGACCCTGATGCACTTTC	Cloning the *AfLFY*
AfLFY–R	GGTGTTGGTCATTTGCTCTTTGGTACCAT	
qAfLFYF2	TGATCCAAGTTCAGAACAATG	Expression analysis of *AfLFY*
qAfLFYR3	CAAGACAATGAAGCGCGTAAC	
Actin 1	ATCTGGCATCACACGTTTTACAA	Expression reference
Actin 2	TCTCACGATTGGCTTTTGGAT	
Ntactin F	CATTGTGCTCAGTGGTGG	Expression reference in tobacco
Ntactin R	AAGGGATGCGAGGATGGA	
CO F	GCAGCAACAACTGGGCAAA	Expression analysis of CO in tobacco
CO R	TTCACACGCCTCGCAAAC	
SOC1 F	CAGATGTGGAGACTGAATTGT	Expression analysis of *SOC1* in tobacco
SOC1 R	CCAGTACAAATCATCTCAGAA	
AP1 F	TAACACAGCCCTTAAGCTCTC	Expression analysis of *AP1* in tobacco
AP1 R	TTAAGATGGCGAAGCATCCAT	
FT F	CCAGCAACTACAGATACAAAG	Expression analysis of *FT* in tobacco
FT R	TTCTGACGCCAACCTGGTG	

The full *AfLFY* gene was amplified from genomic DNA using long PCR with primers LK and LB, as previously described^57^. PCR products were subcloned and sequenced as described above.

### Sequence analysis

BLAST online searches were used to confirm that sequences from selected clones were *LFY* homologs. Predicted protein sequences encoded by *LFY* homologs were retrieved from GenBank (http://www.ncbi.nlm.nih.gov/entyez/query.fcgi and http://blast.ncbi.nlm.nih.Gov/Blast.cgi) and were used to confirm sequence identification and perform phylogenetic analysis. Amino acid sequences were aligned using Geneious 9.0 and a neighbor-joining tree was constructed in MEGA 6 using Kimura two-parameter distances and pairwise deletion of gaps.

### Quantitative real-time PCR expression analysis

The temporal and spatial expression patterns of *AfLFY* were examined during vegetative and reproductive growth. Total RNA was extracted from roots, stems, leaves, and shoot apical meristems during vegetative development and roots, stems, leaves, inflorescence shoot apices meristems (reproductive bud), 1 mm flower buds (flower bud), 5 mm flower buds (alabastrum) and fully opened flowers during the reproductive phase. RNA (1 μg) was reverse transcribed to generate cDNA using a Revert Aid First Strand cDNA Synthesis kit (Thermo, USA). Quantitative real-time PCR was performed in 20 μL reaction volumes containing 2.0 μL of a 1:5 dilution of the cDNA, 1.0 µL of each primer, 10 μL of SYBR Green Master Mix (Applied Biosystems) and 6.0 μL of ddH_2_O. The qPCR conditions were as follows: denaturation at 55 °C for 2 min, 95 °C for 2 min, followed by 40 cycles at 95 °C for 15 s, 60 °C for 1 min on ABI 7500 System thermocycler (Applied Biosystems). The specificity of the amplified product was verified by a melting curve from 60 to 95 °C. Specific primers for qRT-PCR analysis of *AfLFY* (primers qAfLFYF2 and qAfLFYR3) were designed using Primer Express 3.0 (Table 1). Actin was used as an internal reference for normalization of relative expression levels of selected genes. The primers used were actin1 and actin2^34^ (Table 1). Three technical replicates were performed for each sample. The 2^−ΔΔCt^ method was used to calculate relative gene expression^58^.

### Subcellular localization and transient expression

Subcellular localization of AfLFY was examined using transient expression in onion epidermis. The *AfLFY* coding sequence without the termination codon was amplified with primers harboring BamH I and Kpn I restriction sites, and it was inserted into the pBI121-EGFP vector to generate the expression vector pBI121-AfLFY-EGFP. *Agrobacterium tumefaciens* carrying the pBI121-AfLFY-EGFP plasmid was inoculated into 50 mL of LB liquid medium. Onion epidermis was prepared and transfected as described previously^59–61^. After incubation in darkness for 14 h, the onion epidermis was collected and washed, placed on a slide, and observed under a confocal laser microscope (Leica TSP5) with excitation at 488 nm wavelength to monitor EGFP expression.

### Construction and transformation of *AfLFY* in tobacco

The *AfLFY* coding sequence was amplified with primers harboring BamH I and Kpn I restriction sites, and it was inserted into the pBI121 vector to generate the expression vector pBI121-*AfLFY* to examine its biological function. The pBI121-*AfLFY* was transformed into *N*. *tabacum* using leaf discs as described previously with the help of Agrobacterium strain EHA105^62^, and it was cultured in a series of MS media with antibiotics. The rooted transformants were planted in soil and grown in long day (LD) (16 h light/8 h dark) conditions.

### Identification and phenotype analysis of transgenic tobacco plants

Fresh leaves were used to extract genomic DNA and total RNA from wild-type and transgenic tobacco plants to perform genome PCR, RT-PCR and qRT-PCR to verify transformation of plants. The PCR reactions were performed with the *AfLFY* gene-specific reverse primers. To examine the expression levels of *CO*, *FT*, *AP1*, *SOC1* in the transgenic tobacco lines, transgenic plants were sampled for qRT-PCR analysis 0-, 7-, 14-, 21-, and 28 days after planting in soil. The actin gene of tobacco was used as an internal control. Specific primers were designed for *CO*, *FT*, *AP1* and *SOC1* according the tobacco sequence (Table 1). Time from rooted transgenic tobacco plantlets into an artificial soil to the first flower visible was regarded as flowering time. The date, height and number of leaves for each transgenic line were recorded when the first flower was visible. A minimum of three independent samples were conducted for each analysis. SPSS software was used to perform the statistical analyses.

## Results

### Cloning and sequence analysis of *AfLFY*

**Figure 1:**
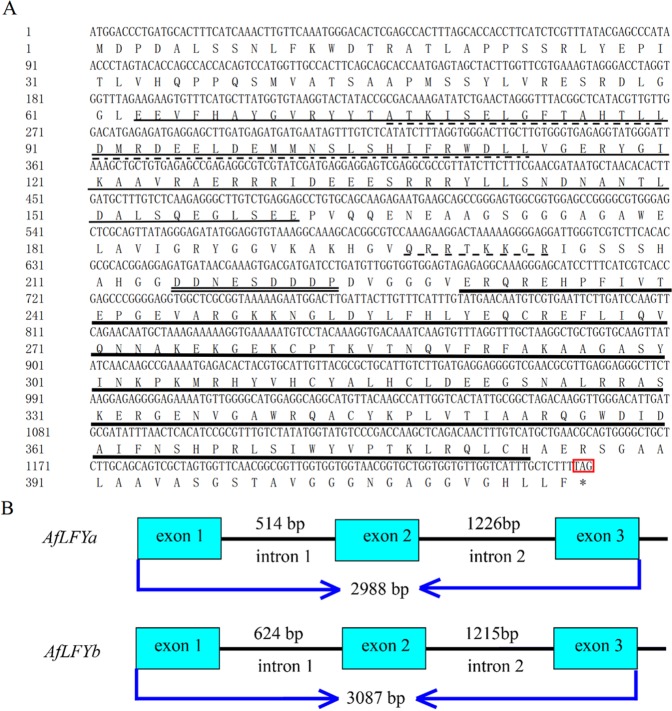
Structure of *AfLFY* in *A*. *frutescens*. (**A**) Nucleotide sequence of the open reading frame of *AfLFY* and the resultant amino acids. The N-terminal conserved region is indicated by a thin solid line; leucine zipper motif is indicated by a dotted line; the basic region is indicated by a dashed line; the central acidic region is indicated by a double solid line; and the C- terminal conserved region is indicated by a thick solid line. (**B**) Structure of the full-length *AfLFYa* and *AfLFYb* gene. Exons are indicated by black boxes and introns are represented by thin lines. Numbers indicate the size of each fragment. Start (ATG) and stop (TAG) codons are shown.

**Figure 2:**
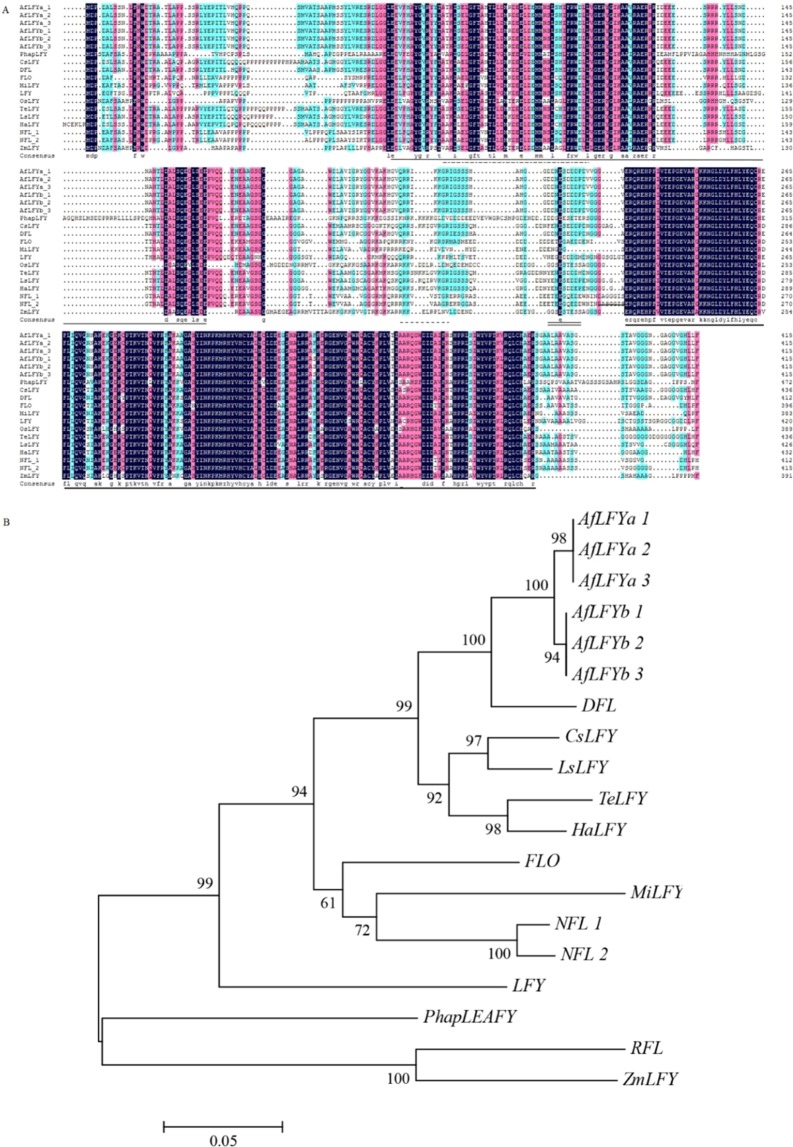
Comparison of AfLFY with LFY homologs. (**A**) Amino acid sequence alignment of AfLFY and plant LFY proteins, the conserved region and typical motif of *LFY* were indicated same as in Fig. 1. (**B**) Phylogenetic analysis of plant LFY amino acid sequences. *PhapLFY*, *Phalaenopsis aphrodite* (KP893636), *CsLFY*, *Cynara scolymus* (XP_024970576.1), *DFL*, *Chrysanthemum lavandulifolium* (AAT51708.1), *FLO*, *Antirrhinum majus* (AAA62574.1), *MiLFY*, *Mangifera indica* (ADX97320.1), *LFY*, *Arabidopsis thaliana* (AAM27931.1), *RFL*, *Oryza sativa* (BAA21547.1), *TeLFY*, *Tagetes erecta* (AEG88962.1), *LsLFY*, *Lactuca sativa* (XP_023744034.1), *HaLFY*, *Helianthus annuus* (XP_021984216.1), *NF1*, *Nicotiana tabacum 1* (AAC48985.1), *NF2*, *Nicotiana tabacum 2* (AAC48986.1), and *ZmLFY*, *Zea mays* (ABC69153.1).

The full-length cDNA of the *LFY*-like gene was successfully amplified and cloned using primers designed against *LFY* homologous sequences. Two haplotypes were obtained among eight positive sequences. There was more than 98% identity between these two sequences. The 2 haplotypes had the same length and only had some differences in nucleotide composition. Both sequences were 1,248 bp in length and encoded 415 amino acids. (GenBank accession number: MK990596, MK990597, named *AfLFYa* and *AfLFYb*). The AfLFY protein contained the typical LFY domain: a leucine zipper, an alkaline region rich in arginine and lysine, and a central acidic region, which were also found in FLO/LFY proteins from other seed-bearing plant species (Fig. 1A). Two distinct copies of *AfLFY* (3024 bp and 3,123 bp) were also found in the *A*. *frutescens* genome using genomic PCR, both of which contained three exons and two introns (MG973291–MG973296, Fig. 1B). Comparison of the predicted AfLFY protein sequences with those of other FLO/LFY-like proteins showed that sequence identity between AfLFY and other LFY homologues ranged from 57% to 93%. Among them, AfLFY shared 72% identity with FLO from *Antirrhinum majus*, 58% with LFY from *Arabidopsis*, and 93% with DFL from *C*. *lavandulifolium* (Fig. 2A). These results confirmed that the sequences isolated from *A*. *frutescens* were *LFY* homologs. Predicted LFY amino acid sequences were used for phylogenetic analysis to determine the evolutionary relationship between AfLFY and other LFY-like proteins (Fig. 2B). Two main clades were apparent, representing monocotyledonous and dicotyledonous species. Sequences from the same taxa were clustered together. The sequences from Asteraceae species were clustered into the dicotyledonous group and were then further clustered into a subclade with a 99% bootstrap support value that was consistent with biological evolution. AfLFY was most closely related to DFL from *C*. *lavandulifolium*.

### Expression of *AfLFY* in *A*. *frutescens*

**Figure 3:**
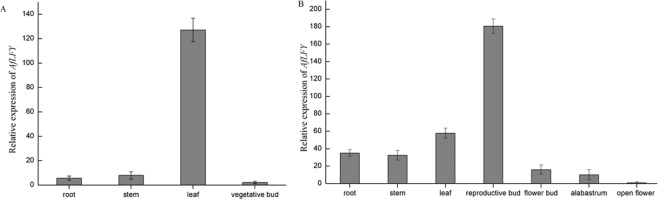
Expression analysis of *AfLFY* and *AP1* using qRT-PCR. Relative expression was assessed in different tissues during (**A**) vegetative growth and (**B**) reproductive growth. Error bars represent ± SD.

The transcription of *AfLFY* in different tissues of *A*. *frutescens* at the vegetative stage and reproductive stage was investigated using qRT-PCR. *AfLFYa and AfLFYb* showed identical expression patterns. For the discussion below, we only present results obtained from *AfLFYa*. *AfLFY* expression was observed in all tested tissues, namely, roots, leaves, stems, shoot apical meristems, and flower buds (Supplementary Fig. S1). During vegetative growth, *AfLFY* was most highest expressed in young leaves followed by stems and roots, with weak expression in vegetative shoot apices (Fig. 3A). During reproductive development, the highest levels of *AfLFY* expression were detected in inflorescence shoot apices meristem. *AfLFY* expression decreased gradually during flower development, with minimal expression observed in fully open flowers (Fig. 3B).

### Subcellular localization of AfLFY

**Figure 4:**
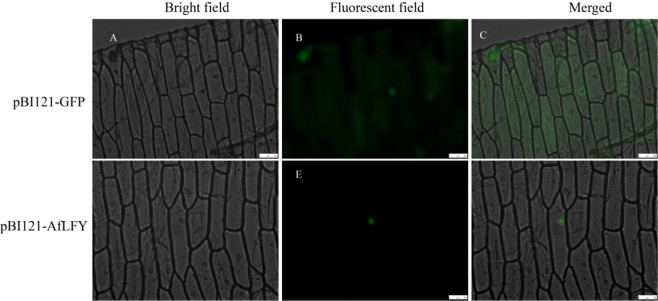
Subcellular localization of AfLFY. EGFP-tagged AfLFY protein was transiently expressed in onion epidermal cells and visualized using fluorescence microscopy. (**A**–**C**) pBI121-EGFP (control), (**D**–**F**) pBI-121-AfLFY-EGFP. Left to right: bright field imaging, fluorescent imaging, and merged image.

Subcellular localization of AfLFY was examined using an EGFP-tagged fusion protein. As there was high identity between the two *AfLFY* sequences, the expression vector pBI121-*AfLFYa*-EGFP was constructed and introduced into onion epidermal cells using *Agrobacterium*-mediated transformation. EGFP expression was examined using fluorescence microscopy after 12–14 hours of incubation in the dark. The pBI121-AfLFYa fusion protein localized only to the nucleus; in contrast, the EGFP control was localized to the nucleus, cytoplasm, and cell membrane (Fig. 4).

### Phenotypic analysis of ectopic expression of the *AfLFY* gene in *N*. *tobacum*

**Figure 5:**
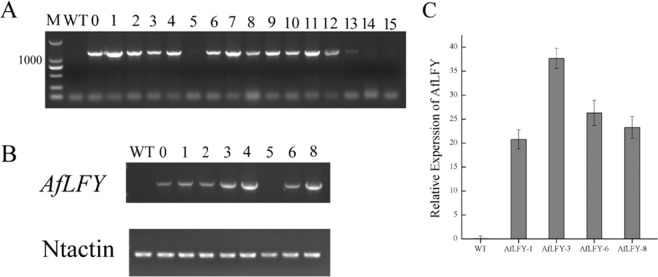
Transgenic plantlet identification. (**A**) Genomic PCR; (**B**) RT-PCR; (**C**) *AfLFY* expression in the four transgenic lines and the wild-type as identified by qRT-PCR. Uncropped gels shown in Supplementary Fig. S2.

**Figure 6:**
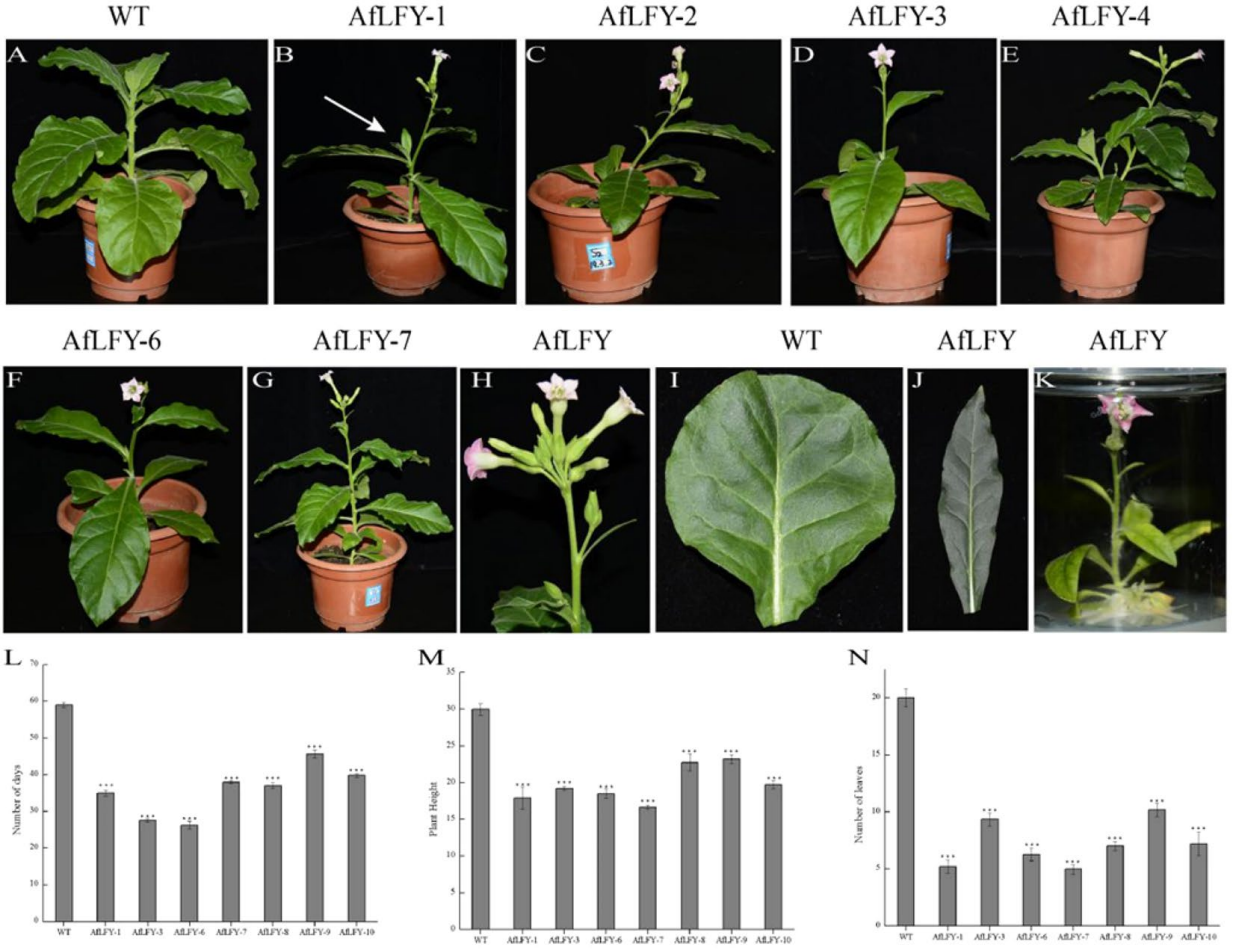
Phenotypic analysis of tobacco overexpressing *AfLFY* and wild-type tobacco. (**A**) The wild type plant (**B**) transgenic line, the arrow indicates the branches produced from the lower leaf axils. (**C**–**G**) Other transgenic lines. (**H**) The flowers were generated from the leaf axils. (**I**)Wild-type tobacco leaf. (**J**)The transgenic line. (**K**) Plantlets flowered on MS medium. (**L**) Days to the first flower opening. (**M**) Plant height at the time of first flower opening. (**N**) The number of mature leaves formed at the time of first flower opening.

To explore the function of *AfLFY* in flower development, an overexpression construct with *AfLFYa* under the control of the CaMV 35 S promoter (35 S::*AfLFY*) was introduced into wild-type tobacco. Approximately 51 independent transgenic tobacco lines were obtained after rooting on MS medium containing kanamycin and rifampicin, and they were verified by genomic PCR, RT-PCR and qRT-PCR (Fig. 5; Supplementary Fig. S2). The resistant plantlets and the wild-type plants were then transferred into pots and grown in an illuminated incubator. Compared with the wild-type (Fig. 6A), all of the transgenic lines led to early flowering and showed obvious changes in flowering time (Table 2; Fig. 6B–H). The earliest flowering observed in transgenic plants overexpressing the *AfLFY* gene occurred 33 days earlier than that of the wild-type plants (Table 2). In addition, ectopic expression of *AfLFY* in transgenic tobacco produced more branches from the axillary and converted all lateral meristems into terminal flowers (Fig. 6B,H). Furthermore, we observed solitary flowers from unrooted shoots cultured on agar-solidified medium (Fig. 6K). Overexpression of *AfLFY* in tobacco also resulted in obvious changes in morphology, e.g., the tobacco leaf shape changed from circular to ovalar (Table 2; Fig. 6I,G), there was a shorter vegetative phase with fewer leaves, and they were shorter (Table 2; Fig. 6M,N).

**Table 2:** Phenotypic analysis of tobacco overexpressing *AfLFY* and wild-type in tobacco.

Sample	stem width/mm	number of leaves/piece	plant height/cm	number of days/day
WT	9.39 ± 0.97941	20 ± 0.79057	30 ± 0.79057	59 ± 0.66708
1	4.49 ± 0.89958***	5.2 ± 0.57009***	17.9 ± 1.51658***	35 ± 0.79057***
3	3.8833 ± 0.24664***	9.3333 ± 0.57735***	19.2333 ± 0.25166***	27.5 ± 0.5***
6	5.574 ± 0.82984***	6.26 ± 0.56391***	18.5 ± 0.64031***	26.2 ± 1.03682***
7	5.7375 ± 1.26516***	4.975 ± 0.4113***	16.675 ± 0.27538***	38 ± 0.40825***
8	6.44 ± 0.84587***	7.02 ± 0.39623***	22.74 ± 1.17601***	37 ± 0.79057***
9	6.61 ± 0.48913***	10.16 ± 0.59414***	23.2 ± 0.54314***	45.72 ± 1.05688***
10	5.874 ± 1.42131***	7.2 ± 1.03682***	19.7 ± 0.54314***	39.8 ± 0.52915***

The values represent the mean ± SD errors. ***Indicates significant differences at P = 0.05. The data for each genotype were measured from five to ten individual plants.

**Figure 7:**
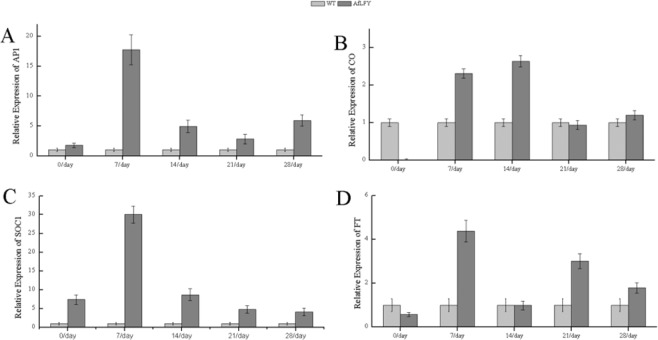
Expression analyses of flower related genes in *AfLFY* transgenic tobacco seedlings grown for days 0, 7, 14, 21, 28 in long-day conditions. (**A**) *LFY* expression. (**B**) *AP1* expression. (**C**) *SOC1* expression. (**D**) *FT* expression. (E) *CO* expression. Error bars represent ± SD.

To determine the relationships of *AfLFY* with other flowering related genes, we analyzed *FT*, *SOC1*, *AP1* and *CO* transcript levels in different developmental stages of transgenic plants. *AP1*, *FT*, and *SOC1* were activated by the ectopic expression of *AfLFY* and were expressed highest at day 7 after planting in soil (Fig. 7A,C,D). However, the expression level of *CO* showed only a subtle increase compared to that of wild-type plants (Fig. 7B).

## Discussion

This study identified an *LFY* homolog in *A*. *frutescens*. Comparisons of predicted protein sequences showed that *AfLFY* belonged to the FLO/LFY superfamily and contained two highly conserved regions at the N- and C-termini. *AfLFY* contained three exons and two introns, which was consistent with other *FLO/LFY* homologs^58,63,64^ and revealed the evolutionary conservation of the *LFY* gene structure and *LFY* function in plants.

The majority of seed-bearing plants examined to date contain one copy of *LFY* in their genomes^65^. However, multicopy genes have been found in some polyploids (e. g., eucalyptus, Monterey pine, and chrysanthemum)^30,36,63,66,67^, suggesting that *LFY* gene has experienced ancient transient duplications events, and that duplicated paralogues were promptly lost in most land plants thus maintaining *LFY* as a single copy^65^. In our study, two haplotypes were identified in six clones from *A*. *frutescens*; they shared more than 94% identity and were in the same clade, which indicated that gene flow might occur in *A*. *frutescens*. The lengths of the three exons were consistent between the two sequences (469 bp, 383 bp, and 396 bp), but intron lengths differed (Fig. 1B). The lengths of intron 1 and intron 2 in *A*. *frutescens* were longer than the length of those introns in Arabidopsis *LFY* (470 bp, 910 bp)^12^, suggesting a rich variety of *LFY* introns among species.

Transcription analysis showed that *AfLFY* was abundantly expressed in inflorescence shoot apical meristems during reproductive development, suggesting a role for *AfLFY* in the transition from vegetative to reproductive development as with *LFY* homologs from other species such as Arabidopsis, chrysanthemums and orchids^16,34,68^. However, *AfLFY* expression was minimal in vegetative shoots and fully opened flowers (Fig. 3A,B). *AfLFY* expression levels were also highly expressed in the leaves during vegetative growth, but they decreased as the plant became mature, suggesting an important role for *AfLFY* in leaf development. A similar role for *LFY* during leaf development was proposed as a result of mutation analysis in legumes and tomato plants^42,43,69^. These results showed that the expression pattern of *AfLFY* diversified in *A*. *frutescens* and varied among different tissues and developmental growth phases.

Significant changes in the expression patterns of *LFY* homologs were observed in different plant. The *LFY* gene in *Arabidopsis* was weakly expressed in the leaf primordia and strongly expressed in the floral meristem and floral organ primordia, but not in the inflorescence meristem^12^. A pattern for *LFY* genes similar to that in Arabidopsis was found in *Antirrhinum*, except that the transcripts of *FLO* were not detected in the stamen primordia^11^. In cucumbers, *CsLFY* was detected in SAM, FM and floral organ primordia^70^. In orchids, *PhapLFY* was strongly expressed in developing inflorescences and leaves during vegetative stage^16^. In hickory, *CcLFY* was strongly expressed in flower buds and leaves, weakly expressed in the stem and showed no expression in the roots^71^. The transcripts of the *LFY* gene in Gerbera were absent from vegetative tissues and were shown to be restricted to young capitula with emerging flower primordia^41^. In our data, *AfLFY* was strongly expressed in the inflorescence shoot meristem and young leaves during the vegetative state. *AfLFY* was also detected in roots, stems, leaves and young flower buds. This various expression pattern in the *LFY* homologs suggests the existence of a functional divergence and differentiated regulatory mechanisms associated with the flowers of different plants^33,65^.

Transient expression of EGFP-tagged AfLFY in onion epidermal cells revealed localization to the nucleus. This suggested that *AfLFY* acted as a transcription factor, which is consistent with previous studies that proposed a transcriptional regulatory role for *AfLFY* in the developing flower^29,35,39,70^.

To explore the function of *AfLFY*, the 35 S::*AfLFY* construct was introduced into tobacco. All transgenic plants showed obvious phenotypes of early flowering and converted the lateral meristems into terminal flowers. These results were consistent with previous studies, showing *LFY* homologs have the ability to regulate floral meristem identity and promote flowering time and cell proliferation^15,32,66,72–75^. In addition, the number of leaves, leaf shape and height of transgenic plants changed (Fig. 6), which correlated with the strong expression of *AfLFY* in vegetative leaves of *A*. *frutescens*. These phenotypes were also observed in other *LFY-like* genes used in the generation of transgenic plants^32,46,76^. Thus, our results suggested that *AfLFY* displays a conserved function in regulating flowering and plays a key role in leaf development during vegetative growth in *A*. *frutescens*.

Previous studies showed that *LFY* together with *AP1*, *FT*, *SOC1* merged the signals from multiple pathways to determine the identity of floral meristems and regulate the flowering time^4,54,77–80^. *SOC1* acted downstream of *FT* in the shoot apex of Arabidopsis^7^. In our study, the expression of endogenous genes *AP1*, *SOC1*and *FT* were upregulated in overexpressing *AfLFY* transgenic tobacco compared to what was observed in wild plants, suggesting that these genes were activated by *AfLFY* and led to early flowering. Thus we speculate that the function of these genes are partially redundant in promoting expression of each other, and then promoting flowering. These results indicate that *AfLFY* plays a central role in the flowering regulatory network. No obvious changes in *CO* expression were observed in transgenic plants compared with wild-type plants (Supplementary Fig. S3), which suggested that *LFY* might work downstream *CO*, and does not feedback regulate *CO* as observed in litchi and gloxinia’ for *CO* is commonly known as key upstream regulator in flowering time, while *LFY* works rather last step in vegetative to reproductive growth transition^77,81^. Further studies on determine the relationship between *AfLFY a*nd other flower related genes will give us more clues to better know the floral development process.

The mechanisms underlying floral development in *A*. *frutescens* are poorly understood. In this study, two copies of an *LFY* homolog, *AfLFY*, were identified in the *A*. *frutescens* genome. Transcriptional analysis showed that *AfLFY* was abundantly expressed in leaves during vegetative growth and in inflorescence shoot apices during reproductive growth, suggesting an important role for *AfLFY* in leaf and inflorescence development. Ectopic expression showed obvious phenotypes of precocious flowering and morphological alterations. Our findings suggested that the *AfLFY* gene plays a vital role in promoting flowering and leaf development in *A*. *frutescens* and might be one of the most important and last step in in vegetative to reproductive growth transition. It would be theoretical basis for foundation of key regulators work upstream. Further studies to determine target genes of *AfLFY* and transgenic analyses in *A*. *frutescens* would be helpful in elucidating the functions of *AfLFY* in regulatory networks.

## Supplementary information

Supplementary Information.
