# Supplementary material for: AfLFY, a LEAFY homolog in Argyranthemum frutescens, controls flowering time and leaf development
Source: Sci Rep. 2020 Jan 31;10:1616. doi: 10.1038/s41598-020-58570-x (PMC6994665; doi:10.1038/s41598-020-58570-x)
Supplement: Supplementary file 1 — Supplementary Information. [file 41598_2020_58570_MOESM1_ESM.pdf]

AfLFY, a *LEAFY* homolog in *Argyranthemum frutescens,* controls flowering time and leaf development

Jing Hu, Qi Jin, Yueping Ma*

College of Life and Health Sciences, Northeastern University, Shenyang, 110004, China

* Corresponding author: Yueping Ma, College of Life and Health Sciences, Northeastern University, Shenyang, 110004, E-mail: mypluna@sina.com

Supplementary legends

Fig. S1. Expression patterns of *AfLFY* in *A. frutescens*, all the vegetative tissues used were collected from the plants in reproductive state except vegetative bud.

Fig. S2 Uncropped gels about transgenic plantlet identification. (A) Genomic PCR; (B)-(C) RT-PCR;

Fig. S3. Expression analyses of flower related genes in *AfLFY* transgenic tobacco.

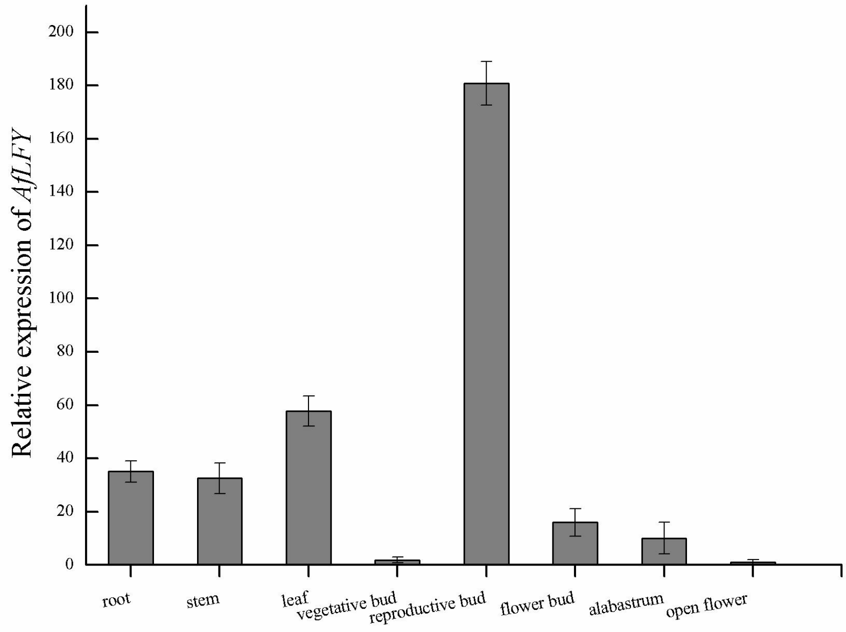

Relative expression of AfLFY
200
180
160
140
120
100
80
60
40
20
0
root
stem
leaf
vegetative bud
reproductive bud
flower bud
alabastrum
open flower

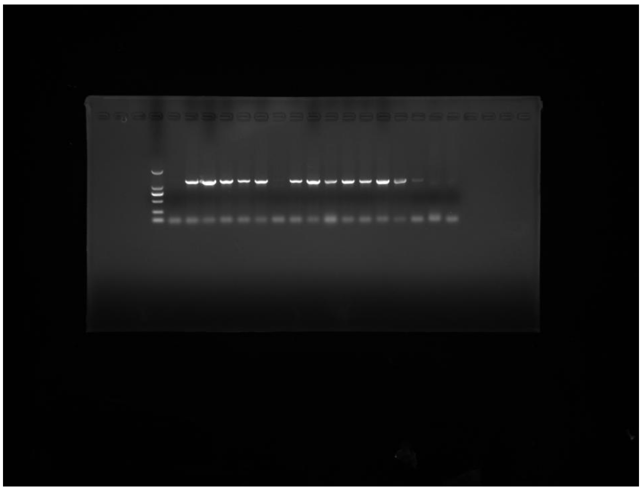

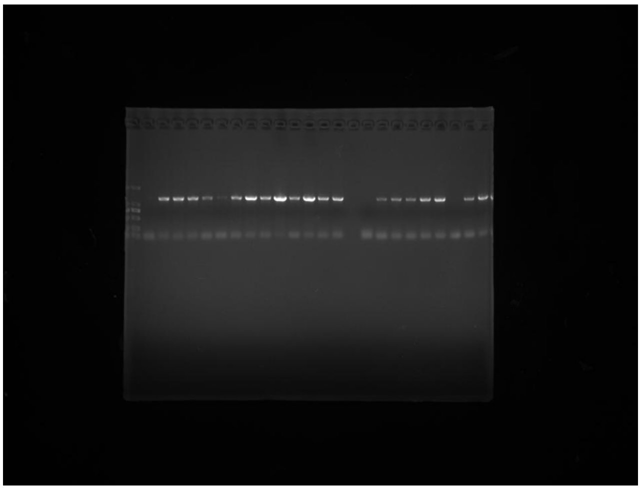

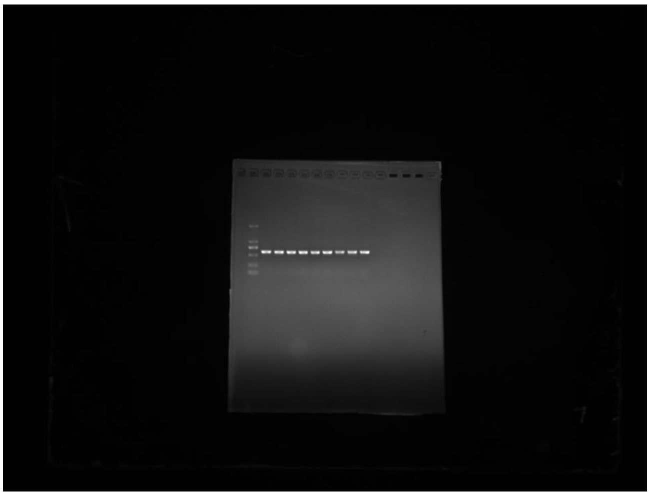

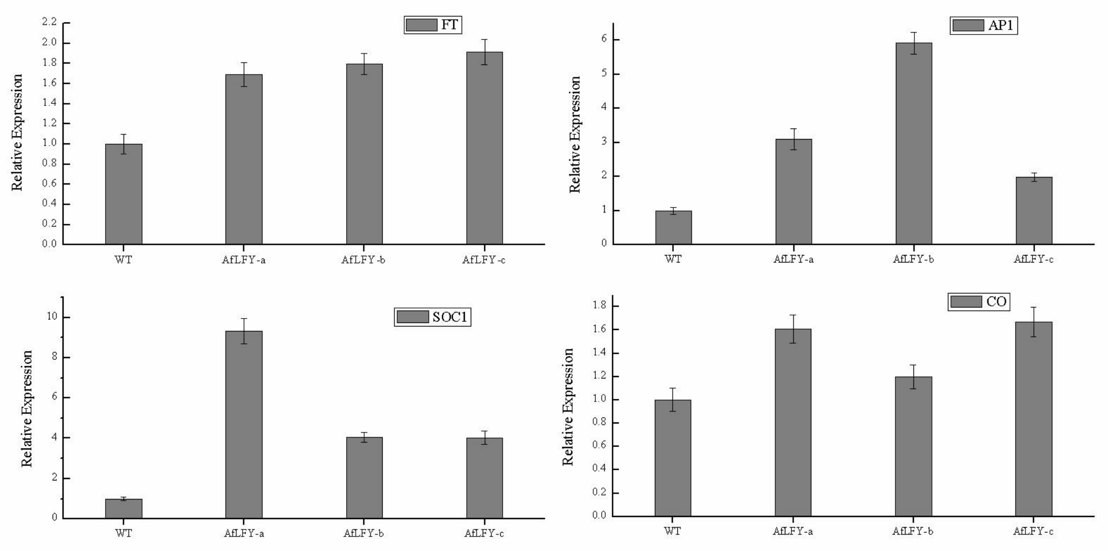
FT
Relative Expression
WT
AfLFY-a
AfLFY-b
AfLFY-c
AP1
Relative Expression
WT
AfLFY-a
AfLFY-b
AfLFY-c
SOC1
Relative Expression
WT
AfLFY-a
AfLFY-b
AfLFY-c
CO
Relative Expression
WT
AfLFY-a
AfLFY-b
AfLFY-c
